# Supplementary material for: Proteomic analysis of Sarcoptes scabiei reveals that proteins differentially expressed between eggs and female adult stages are involved predominantly in genetic information processing, metabolism and/or host-parasite interactions
Source: PLoS Negl Trop Dis. 2022 Dec 6;16(12):e0010946. doi: 10.1371/journal.pntd.0010946 (PMC9725168; doi:10.1371/journal.pntd.0010946)
Supplement: Attachment — Submitted filename: Rejoinder_TW2_rbg1_rbg2_rbg3.docx [file pntd.0010946.s007.docx]

**Editors of PLoS Neglected Tropical Diseases**

**Professor Aysegul Taylan Ozkan and Dr Jo Middleton**

13 September 2022

**Dear Professor Aysegul Taylan Ozkan and Dr Jo Middleton,**

**RE: Ms. Ref. No.: PNTD-D-22-00426_R1 entitled “Proteomic analysis of *Sarcoptes scabiei* reveals that proteins differentially expressed between eggs and female adult stages are involved predominantly in genetic information processing, metabolism and/or host-parasite interactions”**

**We thank you both for handling the manuscript, and the reviewers for their very constructive reports on our manuscript.**

**In the following are our responses in a point-by-point manner (in bold-type):**

**Methods**

*-Are the objectives of the study clearly articulated with a clear testable hypothesis stated?*

*-Is the study design appropriate to address the stated objectives?*

*-Is the population clearly described and appropriate for the hypothesis being tested?*

*-Is the sample size sufficient to ensure adequate power to address the hypothesis being tested?*

*-Were correct statistical analysis used to support conclusions?*

*-Are there concerns about ethical or regulatory requirements being met?*

**Reviewer #1**:

1. Study methods are all very solid and well described in the text. Sample sizes also seem sufficient and statistical analysis is very robust.

**RESPONSE: Thank you.**

2. Can the authors confirm if the adult females selected for the analysis also contained eggs or if care was taken to select non-gravid Af mites in order to prevent cross contamination of egg proteins into the Af proteome?

**RESPONSE: It is not feasible to separate gravid from non-gravid females, such that some females did contain eggs; however, irrespective, there was a differential group of molecules in females *versus* eggs.**

3. Raw data has been deposited in PRIDE but no accession is provided so it is not possible to check this at this stage, a full accession should be provided ahead of publication.

**RESPONSE: Thank you. At this stage, the raw data are only available for peer review. Data access for reviewers was provided within the submitted cover letter (Username: reviewer_pxd032148@ebi.ac.uk; Password: eyBOrRbf). The raw data deposited in PRIDE will become publicly available, and PRIDE ID will be updated when/if the manuscript is accepted.**

**Reviewer #2**:

The study methodology is well mentioned. The objective of the study is well described and hypothesis is novel. Although the sample size calculation is not mentioned, the nature of the study and the number of the proteins analyzed appears adequate. The statistical analysis used is appropriate.

**RESPONSE: Thank you.**

--------------------

**Results**

*-Does the analysis presented match the analysis plan?*

*-Are the results clearly and completely presented?*

*-Are the figures (Tables, Images) of sufficient quality for clarity?*

**Reviewer #1**:

Results are clearly described but please see General Comments section for a more detail assessment of what is required to add further impact to the manuscript. Figures are good and clearly presented but Figure 2B could use a more obvious colour scheme in order to more clearly show the variation in gene expression between life cycle stages and replicates - perhaps a blue/yellow scheme would be more obvious.

**RESPONSE: Thank you. Figure 2B has been modified accordingly.**

**Reviewer #2**:

The analysis results presented are in the line of the study objective

**RESPONSE: Thank you.**

--------------------

**Conclusions**

*-Are the conclusions supported by the data presented?*

*-Are the limitations of analysis clearly described?*

*-Do the authors discuss how these data can be helpful to advance our understanding of the topic under study?*

*-Is public health relevance addressed?*

**Reviewer #1**:

The conclusions are clearly supported by the results of the study, however I really do feel that more can be made of the analysis by adding further discussion of the individual proteins identified and also through an interaction network to really draw out the pathways and protein connections involved between stages. See General Comments section for further details

**RESPONSE: Thank you for the constructive suggestions. We have modified and enhanced aspects of the discussion regarding protein families/groups of interest and interaction networks have been included (page 9, lines 221-228; page 13-14, lines 339-425; tracked).**

**Reviewer #2**:

The conclusion presented is more like a summary. It can be modified to include their main findings and its public health implications along with limitation and future direction of research.

**RESPONSE: Thank you for the constructive suggestions. We agree, and we have enhanced the conclusion section (see pages 18-19; tracked).**

--------------------

**Editorial and Data Presentation Modifications?**

**Reviewer #1**:

A few minor editorial changes are required as described here:

1. Line 37: "Scabies is a neglected tropical disease......"

2. Line 56: "Scabies is a disease caused by the ectoparasitic mite Sarcoptes scabiei...."

3. Line 93: Remove: "one of the most important parasitic mites of humans and other animals (e.g. pig, dog and wombat) worldwide"

4. Line 98: "defining the proteome of the key developmental stages...."

5. Line 112: I'm not sure that production is the correct terminology for "rearing/maintaining" scabies mites.

6. Line 124: The title of this section is repeated from the previous section and needs to be changed to "Protein extraction and characterisation" or something similar..

7. Line 344: ".. in D. farinae and ...."

**RESPONSE: Thank you. These editorial changes have been made.**

**Reviewer #2**:

None

--------------------

**Summary and General Comments**

**Reviewer #1**:

This is a very well written and well described article providing a really excellent characterisation of the Scabies mite proteome across key reproductive life cycle stages. The study has been conducted very well and the analysis and results presented are robust and clearly add significantly to the existing scientific knowledge of this highly important mite.

However, in my opinion the paper does require some further analysis/exploitation of the existing data before it can be considered suitable for publication in PLOS NTD. In particular more needs to made of the really impressive proteomic dataset that was generated from the study and this should involve at a minimum a protein-protein interaction network, for example as can be achieved with a tool such as StringDB. Whilst I appreciate that Sarcoptes scabiei is not annotated within StringDB then the annotation for a closely related mite such Tetranychus urticae should be used instead. I feel that this analysis will be important to provide further information on the connections between the proteins identified, especially in the context of the differential expression analysis between life cycle stages.

This also comes on to my second point, which is the current lack of in depth discussion around specific protein classes and families that were identified from the differential expression analysis as well as from the standard characterisation of the proteome of the mite. For example, there is little if any discussion around key allergens, i.e. house dust mite homologues, vitellogenins and other proteins involved in reproduction and nutrient provision for the developing embryos and also cuticular proteins and structural muscle proteins, i.e. myosin/troponins etc. Also further discussion is required around the enzyme classes identified, including the scabies mote pseduoproteases, SMIPPs which were identified in the initial analysis. The current manuscript title and the analysis of results presented are underwhelming and I feel that a lot more can be made of this impressive dataset by carrying out the further analysis described above.

Finally, Figure 2 may be further improved by adding hierarchical clustering of proteins at both the sample (column) and protein (row) levels as this may also help to identify clusters of proteins between life cycle stages.

The conclusions section then needs to be made stronger, for example what outcomes from the study will help to identify novel acaricides in the future?

**RESPONSE: Thank you for the constructive suggestions. The suggested protein-protein interaction networking was performed via StringDB using the Cytoscape platform (page 9, lines 221-228, page 13-14, lines 339-425, and Table S6). We have enhanced the discussion of selected protein families/groups (pages 16-17), and the title, Figure 2B, and the discussion and conclusion sections have been modified/enhanced.**

**Reviewer #2**:

The authors have analyzed proteins form S. scabiei to find the relation of such proteins with different developmental stages. The liquid chromatography–mass spectrometry technology was used for detection of such proteins is using. The study methodology is well mentioned and results are well presented. However, the abstract presented can be structured and limitation of the study can be mentioned.

**RESPONSE: Thank you for the constructive suggestions. We have enhanced the abstract (see page 1; tracked). Due to the word limit imposed for the abstract, we cover the possible limitations of this study in the conclusion section (page 18; tracked)**

--------------------

**CONCLUSION**

**Again, we thank the reviewers for their valuable reports, and we hope that we have satisfactorily addressed all of the issues raised. We believe that the revised manuscript (PNTD-D-22-00426_R1) now meets the requirements for publication in PLoS Neglected Tropical Diseases. We look forward to the final decision.**

**On behalf of authors,**

**Tao Wang**
