# Supplementary material for: Proteomic analysis of Sarcoptes scabiei reveals that proteins differentially expressed between eggs and female adult stages are involved predominantly in genetic information processing, metabolism and/or host-parasite interactions
Source: PLoS Negl Trop Dis. 2022 Dec 6;16(12):e0010946. doi: 10.1371/journal.pntd.0010946 (PMC9725168; doi:10.1371/journal.pntd.0010946)
Supplement: Attachment — Submitted filename: Rejoinder_TW.docx [file pntd.0010946.s008.docx]

**Editors of PLoS Neglected Tropical Diseases**

**Professor Aysegul Taylan Ozkan and Dr Jo Middleton**

11 November 2022

**Dear Professor Aysegul Taylan Ozkan and Dr Jo Middleton,**

**RE: Ms. Ref. No.: PNTD-D-22-00426_R1 entitled “Proteomic analysis of *Sarcoptes scabiei* reveals that proteins differentially expressed between eggs and female adult stages are involved predominantly in genetic information processing, metabolism and/or host-parasite interactions”**

**We thank you both for handling the manuscript, and the reviewers for their very constructive report on our manuscript.**

**In the following are our responses in a point-by-point manner (in bold-type):**

**Methods**

*-Are the objectives of the study clearly articulated with a clear testable hypothesis stated?*

*-Is the study design appropriate to address the stated objectives?*

*-Is the population clearly described and appropriate for the hypothesis being tested?*

*-Is the sample size sufficient to ensure adequate power to address the hypothesis being tested?*

*-Were correct statistical analysis used to support conclusions?*

*-Are there concerns about ethical or regulatory requirements being met?*

**Reviewer #3**:

Although no clearly articulated single hypothesis is tested in the paper, this work is an example of a well-prepared, novel study presenting new knowledge essential in human disease studies/epidemiology and general mite biology and parasitology. Nonetheless, the work has an ambitious aim, which has been accomplished. Only the presentation of the problem and the new data obtained provide a source for hypotheses that can be tested in further research, as the authors have suggested in the Discussion. I advise that one sentence be included to make it even more explicit that this work can be (and probably will be) a starting point for further, more detailed, or, i.e., purely experimental studies.

The study design is appropriate to address the stated objectives. Of course, more methods and objectives could be stated, but it is impossible to carry out so many tasks in one study (this statement applies to all studies).

There is no information why exactly such a sample size has been analysed but taking into account the prevalence of parasitic mites in general, difficulties in obtaining a decent number of individuals, etc. The sample size seems to be adequate for the conducted study.

At least, as far as my knowledge and the authors' descriptions of methodological approaches provided, together with references to those commonly used and practised in a similar type of research, allow me to conclude I see no objection to the methods used in this investigation. In addition, the authors provided information and results of the relevant statistical analysis.

The authors supplied information on ethics approval due to the study with animals. It fulfils all the ethical requirements regarding the type of studies conducted.

**RESPONSE: Thank you. We agree that the current work is a starting point for more detailed studies in the future. This is why we emphasised this in the Discussion and Conclusions sections (e.g., lines 444-451).**

--------------------

**Results**

*-Does the analysis presented match the analysis plan?*

*-Are the results clearly and completely presented?*

*-Are the figures (Tables, Images) of sufficient quality for clarity?*

**Reviewer #3**:

The analysis performed during the research and after that presented in the Results section of the manuscript adequately matches the plan outlined in its previous parts.

It is worth noting the thorough presentation of the results, with many references to the extracted data constituting the supplementary material and that deposited in the public database. Also, unexaggerated comparisons and explanations of submitted figures indicate a satisfactory reporting of the exciting findings.

Figures are of sufficient general quality. The data in the tables are precise, mainly when explained directly in the manuscript. However, I must say that:

a) shades of colours in Figure 1B may be challenging to read, especially for people with impaired vision, as some are pretty similar. I would consider changing the colours to obtain better contrast before releasing the potential publication so that the results presented were not questionable,

b) Fig. 2B should be stretched vertically because the branches of the tree are remarkably close together, hence quite unreadable,

c) and the last issue of the graphical representation of biological processes and associated protein pathways in Fig. 4. I use the 4K monitor, and I had a problem seeing them at all at first glimpse. The lines of those pathways should be thicker.

I think that my proposed corrections may improve the readability of graphs for the readers.

**RESPONSE: Thank you. Figures 1B and 2B have been modified accordingly. The current graph of functree in Fig. 4 is very clear. We retained the pathway lines as they associate with the dots that represent individual biological categories/processes and/or KEGG pathways.**

--------------------

**Conclusions**

*-Are the conclusions supported by the data presented?*

*-Are the limitations of analysis clearly described?*

*-Do the authors discuss how these data can be helpful to advance our understanding of the topic under study?*

*-Is public health relevance addressed?*

**Reviewer #3**:

The presented data support conclusions.

    There is much to say about the limitations, although the authors provided some degree of it. The sentence “Originally…” (line 428) would be better if provided as an example of such limitation instead of unsuccessful original plans (maybe you will enrich the topic soon).

    The authors discuss how these data can help advance our understanding of the topic under study.

    The public health relevance is mentioned, but it only makes an impression as a formal addition. Meanwhile, the research presented in this manuscript shows how many exciting things remain to be discovered about the parasitic mite of man, which was probably first mentioned in the literature by Aristotle. I suggest adding something more related to public health here.

**RESPONSE: Thank you. We have modified this sentence (lines 433-434). The current work is a first step to characterise the proteome of *Sarcoptes scabiei* and much more work is still needed to identify and confirm novel intervention targets in this mite; the authors are not willing to overinterpret the findings at this stage.**

--------------------

**Editorial and Data Presentation Modifications?**

**Reviewer #3**:

Lines 68-72. Some reference would be welcomed.

**RESPONSE: Thank you. A reference has been added.**

    Line 87. I think there should be “mite genomic….”

**RESPONSE: Thank you. This has been modified accordingly.**

Line 117. Production might be the correct word for, e.g., pigs if they are sold for meat. Also, even mass production of predatory mites of the family Phytoseiidae is used for natural control of mainly spider mites (Tetranychidae) as they damage many cultivated plants. In this case (even if there are examples in some literature), “production” is an incorrect term, and I advise the word “rearing” instead of the former one.

**RESPONSE: Thank you for the suggestion; however, we disagree with this comment and would like to keep the wording as is.**

    Line 360. Sarcoptes is the genus name. Also, we do not know if the results can be expanded to other species classified to it. Hence, “S. scabiei individuals/representatives/…” sounds better without ambiguities.

**RESPONSE: Thank you. This has been modified accordingly.**

Line 395. For the general reader, it would be helpful to explain what “stratum granulosum” is. The same is true for “cross-talk” (lines 337, 378, 408, 438).

**RESPONSE: Thank you for the suggestions. Both ‘stratum granulosum’ and ‘cross-talk’ are commonly used and comprehensible terms within the context of the work.**

Lines 93, 369, 413. There are mistakes in the specific epithet; please replace “farina” with “farinae.”

**RESPONSE: Thank you. Changes have been modified accordingly.**

--------------------

**Summary and General Comments**

**Reviewer #1**:

The authors have clearly addressed the comments raised in the review and I would now be happy to see this paper published in its current form.

**RESPONSE: Thank you.**

**Reviewer #3**:

The manuscript submitted, in addition to the enormous effort to deliver the research results undertaken therein, is worthy of being considered for publication in the PLOS Neglected Tropical Diseases journal. The mistakes are only nominal, most of them in the form of editorial amendments. However, the copyeditors could not cope with this task given the poorly represented scientific field. Nevertheless, the oversights found, or details that need ONLY MINOR improvements, oblige me to propose a 'Minor review' as my recommendation for the Editor at that moment. My comments and suggestions are intended to ensure that the contributors will publish a paper of the highest possible quality. After correcting and considering a few things already mentioned in my review, the manuscript should be accepted.

I agree to disclose my name to the authors to build better, transparent science that is a core for the PLOS non-profit corporation. I declare that I have no conflict of interest. I congratulate the authors for their excellent work and the Editor for inviting me to review.

**RESPONSE: Thank you.**

--------------------

**CONCLUSION**

**We thank the reviewers for their reports, and we have addressed all of the issues raised. We believe that the revised manuscript (PNTD-D-22-00426_R2) now meets the requirements for publication in PLoS Neglected Tropical Diseases. We look forward to the final decision.**

**On behalf of authors,**

**Tao Wang**
